# A systematic review of guinea fowl diversity and its economic, socio-cultural and ecological significance in Africa

**DOI:** 10.1007/s11250-025-04723-x

**Published:** 2025-11-03

**Authors:** Salifu Fallalu Rahman, Wissem Baccouri, George Wanjala

**Affiliations:** 1 Institute of Animal Sciences and Wildlife Management, University of Szeged, Andrássy út 15, Hódmezővásárhely, 6800 Hungary; 2 Doctoral School of Animal Science, University of Debrecen, Debrecen, 4032 Hungary; 3 Department of Animal Science, Institute of Animal Science, Biotechnology and Natural Conservation, Faculty of Agricultural and Food Sciences and Environmental Management, University of Debrecen, Böszörményi út 138, Debrecen, 4032 Hungary

**Keywords:** Climate change, Food security, Genetic diversity, Phenotypic diversity, Rural households

## Abstract

Guinea fowls (*Numida meleagris*), native to Africa and domesticated over 2000 years ago, provide an important yet underrecognized domestic avian genetic resource. They play a crucial role in enhancing Africa’s rural food security, generating revenue, and sustaining socio-cultural activities in many African countries. This study analyzes existing literature about the genetic and phenotypic diversity of guinea fowl populations in Africa, highlighting the significance of this diversity in improving socio-cultural, economic, and ecological landscape. We contend that the diverse roles guinea fowls play in local economies and cultural practices are largely driven by their extensive phenotypic variation and underlying genetic diversity. Notwithstanding their potential, guinea fowl production is hindered by challenges like inadequate breeding infrastructure, unstructured market infrastructure, and a deficiency in funding initiatives. We propose a systematic, community-based breeding program to enhance productivity and conserve genetic resources. This framework offers practical assistance for policymakers, academics, and breeding organizations aiming to fully utilize guinea fowls for sustainable agricultural advancement in Africa. Furthermore, to enhance guinea fowl breeding in Africa, it is necessary to invest in research on economically important traits and the best strategies to improve them for the benefit of the community.

## Introduction

Guineafowl *(Numida meleagris*) has been an important part of African culture and economy for a long period of time and have been domesticated since ancient times, with the most recent domesticated lineage derived from West African subspecies *N. m. galeata* (Moreki and Radikara 2013). Domestication most likely occurred in Mali and Sudan about 2000 years before present (BP) (Larson and Fuller 2014). Centuries of selective breeding have resulted in significant morphological and behavioural divergence from wild forms, including increased body mass, modified pigmentation, and reduced parental care. Nonetheless, domestic guineafowl retains some ancestral traits such as white facial skin and rounded gape wattles. Archaeological evidence suggests that guinea fowls were present in Egyptian tomb paintings dating as far back as 2400 BC, suggesting their significance in early human societies then (Ayorinde 2004), and in fact the author referred to guinea fowls as “spice of life”.

Their domestication was necessitated because of their ability to provide meat and eggs while requiring almost no care, making them vital for free range or extensive farming systems in harsh climates (Abdallah and Oyebamiji 2024). Presently, domesticated guinea fowls have spread and inhabited several parts of the world, including Europe, Asia and the Americas, where they are now raised for commercial and subsistence farming. For example, France and the USA are known to own some big companies working with guinea fowl genetic improvement and intensive production (Baéza et al. 2001; Araújo et al. 2023).

The production of guinea fowl plays a critical role in addressing poverty alleviation, enhancing socio-economic activities, promoting ecological management, and contributing to food security across Africa. This significance is largely attributed to the high level of phenotypic diversity exhibited by guinea fowls, including considerable variations in plumage color patterns, helmet morphology, and other morphometric traits, which support their adaptability to diverse environmental conditions and market preferences. A key feature of Africa’s economy is that more than 70% of the farming activities are practiced by small-scale households, and most of the livestock agriculture is conducted within extensive farming systems (Kom et al. 2022). This system is prone to the vagaries of climate and is characterized by limited resource input. Therefore, it suffices to say that the adoption of species and breeds that remain productive under such environmental circumstances guarantees sustainable agricultural production.

Guinea fowl production has not been given the attention it deserves, both globally and at Africa’s continental level. There is notably limited research that has been conducted on guinea fowls compared to other domestic livestock species such as chickens, sheep, cattle, and pigs. In fact, recently it has been observed that guinea fowl is not included in the FAOSTAT database (Faostat 2022) as a standalone species. It is possibly counted in the list of “others,” suggesting that this vital species has been ignored since domestication, possibly due to the absence of a well-structured breeding program or a defined guinea fowl product species value chain. At the global scale, geese and guinea fowl together account for nearly 2% of total poultry meat production, with Africa contributing about half of this category, classified as “other poultry” by the FAOSTAT (2023). Nevertheless, some African countries report guinea fowl populations as stand-alone species. For example, in Guinea, guinea fowl represent approximately 7% of the national poultry population (Avornyo et al. 2016), while in Burkina Faso they were estimated at 8.46 million birds in 2018 Traoré et al. (2018). Despite these contributions, up-to-date and comprehensive data on regional and global guinea fowl populations remain scarce, underscoring the need for greater investment in research and development of this species within poultry production system. To enhance understanding and advance guinea fowl breeding efforts in Africa, this review critically examines and synthesizes existing literature on the diversity (genetic and phenotypic) status of guinea fowl populations across the continent. In particular, we explore the significance of this diversity in promoting socio-economic activities and ecological management. Furthermore, we identify major challenges hindering the effective production and utilization of guinea fowls. Based on these insights, we propose a strategic framework aimed at supporting the sustainable breeding, conservation, and utilization of guinea fowl genetic resources in Africa.

## Methodology

We conducted an advanced literature search across three major academic databases, Scopus, PubMed and Web of Science (WoS), to identify relevant sources. The search strategy employed a combination of keywords including “Guinea fowls,” “economic importance,” “ecological importance,” “social importance,” “socio-economic,” “genetic diversity,” “genetic characterization,” “phenotypic diversity,” “phenotypic characterization,” “Africa,” and the names of individual African countries. Boolean operators (“AND,” “OR,” “NOT”) were applied to refine the search results. No restriction was placed on the year of publication, as the review also aimed to examine publication trends over time. The collected literature was analyzed using the Bibliometrix R package (Derviş 2019), implemented within the R statistical software environment (Team 2019). The screening process, including inclusion and exclusion criteria, followed the Preferred Reporting Items for Systematic Reviews and Meta-Analyses (PRISMA) guidelines (Page et al. 2021). Information extracted from the identified sources included genetic diversity indices derived from molecular markers, as well as phenotypic diversity measurements reported across various studies.

## Results and interpretations

### Description of literature sources included in the study

**Fig. 1:**
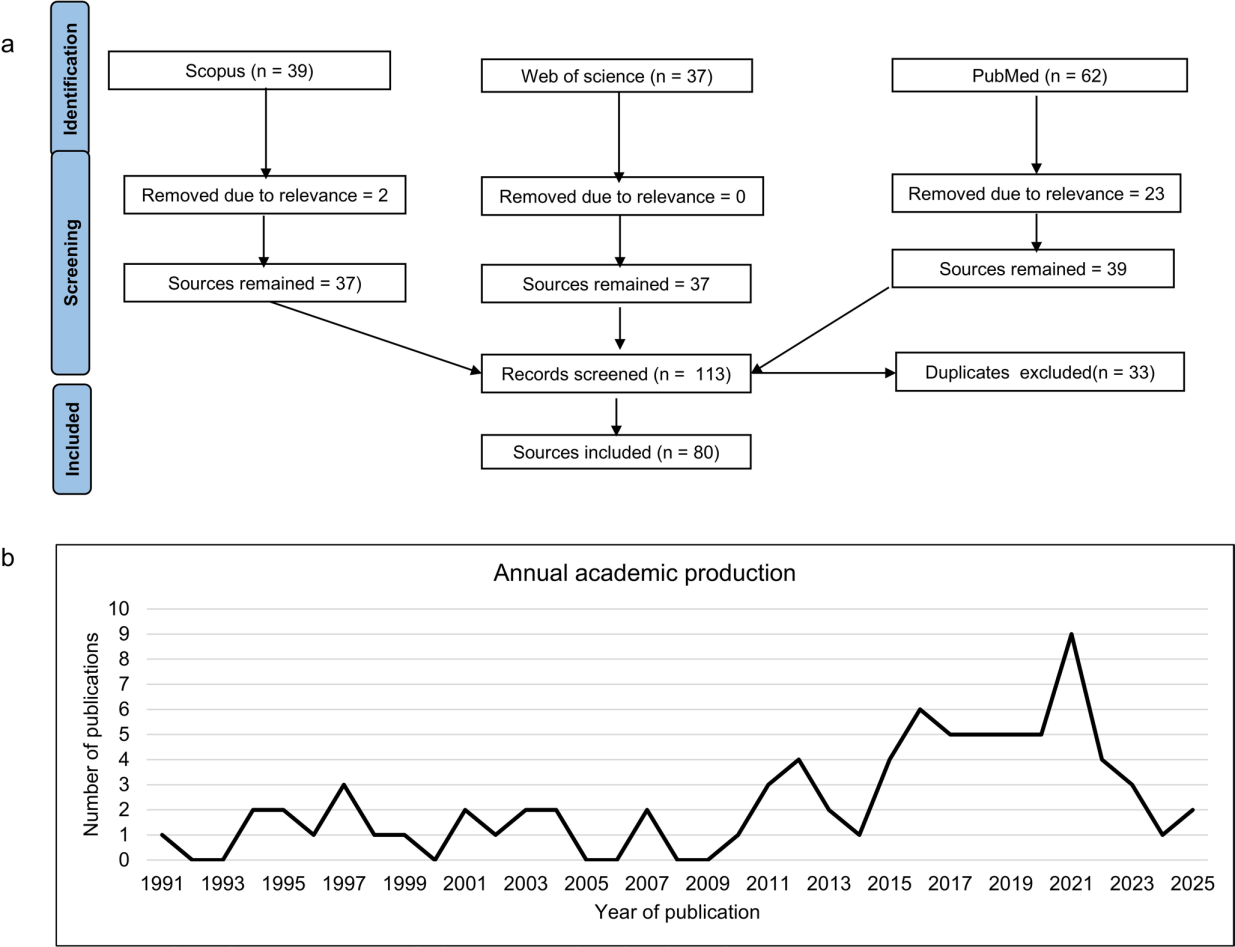
Visualization of: (**a**) PRISMA literature review protocol, (**b**) Publication annual trend

A total of 138 sources were identified across three databases: Scopus (39), Web of Science (37), and PubMed (62). Following screening based on PRISMA guidelines, 80 sources were retained and subsequently subjected to bibliometric analysis. The bibliometric analysis revealed that the selected literature spans from 1991 to 2025, with a total of 80 documents (Fig. 1a) sourced from 62 distinct outlets, including journals, books, and other scientific publications (Table 1). This relatively small body of work, coupled with an annual growth rate of just 2.06% (Fig. 1b), underscores the slow and fluctuating scholarly attention toward guinea fowl research in Africa over the past three decades. In terms of authorship patterns, the study involved 395 unique authors, highlighting a strong collaborative research culture, as evidenced by an average of 6.3 co-authors per document and an international co-authorship rate of 31.25%. This international collaboration indicates a moderately high level of global research engagement, despite the overall limited output. Regarding document types, the majority (*n* = 52) were classified as research articles, with a minor representation of book chapters, data papers, comparative studies, and reviews. This predominance of original research articles suggests that empirical contributions rather than theoretical discussions dominate the field.

**Table 1:** Description of the literature sources included in the study

Description	Results
Main information about data	
Timespan	1991:2025
Sources (Journals, Books, etc.)	62
Documents	80
Annual Growth Rate %	2.06
Document Average Age	11.7
Average citations per doc	5.85
References	0
Document contents	
Keywords Plus (ID)	435
Author’s Keywords (DE)	379
Authors	
Authors	395
Authors of single-authored docs	6
Authors collaboration	
Single-authored docs	9
Co-Authors per Doc	6.3
International co-authorships %	31.25
Document types	
Article	52
Article; book chapter	1
Article; data paper	1
Comparative study; Journal article; Research support, N.I.H., extramural; Research support, U.S. Gov’t, non-p.h.s.	1
Comparative study; Journal article; Research support, non-U.S. Gov’t	1
Comparative study; journal article; Research support, U.S. Gov’t, p.h.s.	1
Journal article	15
Journal article; Research support, N.I.H., Extramural; Research support, non-U.S. Gov’t	1
Journal article; Research support, non-U.S. Gov’t	5
Journal article; research support, non-U.S. Gov’t; Research support, U.S. Gov’t, p.h.s.	1
Review	1

### Genetic and phenotypic characterization of Africa’s guinea fowls

#### Genetic diversity

Genetic diversity plays a critical role in ensuring species adaptability, resilience, and long-term evolutionary potential (Weigend and Romanov 2001; Wanjala et al. 2021). It is essential to distinguish between genetic and phenotypic diversity: genetic diversity refers to variations at the DNA level, while phenotypic diversity pertains to observable characteristics; importantly, one does not directly determine the other. To accurately classify species into breeds, ecotypes, or sub-populations, a comprehensive understanding of their genetic architecture is required, typically achieved through genetic characterization studies. Parallel efforts in phenotypic characterization are also valuable, as they enable the systematic assessment of morphological, physiological, and behavioral traits across and within populations. Together, these approaches provide a robust framework for the conservation, improvement, and management of biological resources.

Genetic characterization has been widely conducted across various domestic livestock species, including sheep (Da Silva et al. 2025), cattle (Mustefa 2023), and chickens (Bodzsar et al. 2009) among others. However, studies specifically focusing on the genetic characterization of guinea fowls, particularly within Africa or its individual countries remain notably scarce, further underscoring the limited scientific attention given to this species.

It is important to recognize that the field of molecular genetics has experienced rapid technological advancements since the development of Restriction Fragment Length Polymorphism (RFLP) in the 1970s (Botstein et al. 1980) to the emergence of next-generation sequencing (NGS) technologies (Metzker 2010). While many earlier technologies have become outdated, NGS offers more comprehensive and high-throughput insights into genetic architectures. Nevertheless, the application of NGS in guinea fowl studies remains limited. Only a few studies employing next-generation sequencing (NGS) technologies on guinea fowl have been identified, including those by Moraa et al. (2025) and Vignal et al. (2019), which primarily focused on populations outside the African continent. However, there is a growing trend in applying NGS technologies to African guinea fowl populations, as evidenced by emerging studies aimed at elucidating the domestication history of the species in West Africa (Shen et al. 2021).

To explore the current genetic diversity status of Africa’s guinea fowls, we relied on results obtained using various genetic markers (Table 2), many of which are now considered outdated. Despite this, we believe that they data provides a reliable representation of the species’ genetic landscape during the periods studied. Moving forward, we strongly advocate for a continent-wide, large-scale genetic characterization initiative, utilizing modern technologies, to generate comprehensive data that will inform evidence-based conservation strategies.

In general, studies have demonstrated that Africa’s guinea fowl populations exhibit high levels of genetic diversity (Orounladji et al. 2023; Traoré et al. 2018; Soara et al. 2022). This finding is particularly significant in the context of the current era of climate change, where sudden and unpredictable environmental shifts pose serious risks to biodiversity. Populations with limited genetic variability are more vulnerable to extinction, as their homogenous genetic makeup may restrict their ability to adapt to new or challenging environmental conditions. Therefore, the high genetic diversity observed among Africa’s guinea fowl populations may serve as a critical buffer, enhancing their resilience and long-term survival prospects.

**Table 2:** Genetic diversity levels of Africa’s guinea fowl populations

Population name	Region	No. of samples	Markers used	Genetic diversity indices			Reference
				Ho	He	HD	
Indigenous guinea fowl	Togo	188	Microsatellite markers	0.512	0.604		(Soara et al. 2022)
Domesticated and wild guinea fowl	Sudan	184	Microsatellite markers	0.364- − 0.494			(Weimann et al. 2016)
Guinea fowl	Benin	96	Microsatellite markers.	0.492	0.595		(Orounladji et al. 2023)
Indigenous guinea fowls	Ghana	168	Microsatellite markers.	0.284–0.426	0.291–0.408		(Agbolosu et al. 2014)
Upper-west region	Ghana	43	Microsatellite markers.	0.459	0.569		(Kayang et al. 2010)
Northern region	Ghana	94	Microsatellite markers.	0.572	0.517		
Legon	Ghana	26	Microsatellite markers.	0.603	0.441		
Benin	Benin	27	Microsatellite markers.	0.518	0.532		
Helmeted Guinea Fowl	Nigeria	215	mtDNA			0.693 ± 0.022	(Adeola et al. 2015)

Ho: Observed homozygosity, He: Expected heterozygosity, HD: Haplotype diversity, mtDNA: Mitochondrial DNA

#### Phenotypic diversity

The phenotypic diversity exhibited by African guinea fowl populations reflects a complex interplay of genetic, socio-cultural, and ecological management factors. This diversity manifests in a broad range of morphological traits and adaptations, underlining the critical role of both molecular and traditional morphological studies in comprehensively understanding population structures.

Although genetic diversity assessments have revealed high levels of heterozygosity among various guinea fowl populations across Africa, it is the phenotypic diversity observable through morphological and biometric variations that provides practical insights into adaptation and survival strategies. For instance, research conducted in northern Togo employing microsatellite markers indicated significant genetic variation among guinea fowl populations, which was consistent with notable phenotypic differentiation across subpopulations in West Africa and Sudan (Soara et al. 2022). Complementing these findings, Weimann et al. (2016) reported a clear genetic and phenotypic distinction between domesticated and wild guinea fowls in Sudan, suggesting that domestication processes have narrowed genetic and morphological variability among domestic stocks compared to their wild counterparts. These observations affirm that reductions in genetic diversity through domestication are often reflected in constrained phenotypic variation.

Morphometric analyses further highlight the substantial phenotypic diversity across different ecological settings in Africa. In Cameroon, Dongmo et al. (2023) documented considerable variability in morphometric traits, including body measurements, indicative of local adaptation to varying environmental pressures. Similarly, studies from Nigeria emphasized the presence of distinct morpho-biometric traits among indigenous guinea fowl populations, such as differences in body size and weight, largely influenced by regional environmental conditions and management practices (Yakubu et al. 2022). Such phenotypic distinctions are crucial for designing effective breeding programs and improving local farming systems.

Additional dimensions of phenotypic diversity, such as feather coloration patterns, have been linked to underlying genetic variation. The occurrence of diverse plumage types, including pearl grey and white varieties, illustrates the outcomes of selective breeding and local cultural preferences (Brown et al. 2017). These phenotypic traits are not merely of aesthetic or commercial interest but are also central to understanding adaptive potential and market value (Abdul-Rahman et al. 2019). Moreover, socio-economic studies, such as those conducted in Benin, emphasize the necessity for integrated genetic and phenotypic research to enhance guinea fowl productivity and conservation strategies (Orounladji et al. 2023). Taken together, the breadth of phenotypic diversity observed across Africa’s guinea fowl populations underscores the importance of holistic approaches that integrate genetic, morphometric, and socio-economic dimensions in conservation and improvement initiatives.

In many domestic livestock species, genome-wide association studies (GWAS) and investigations into signatures of selection have been extensively utilized to elucidate the genetic underpinnings of phenotypic variability. For example, in cattle, the *POLLED* locus, encompassing the *PC* and *PF* variants, has been identified as responsible for the hornless (polled) phenotype (Medugorac et al. 2012). In sheep, mutations in the *RXFP2* gene regulate horn development (Kijas et al. 2012), while in goats, deletions within the *PISRT1* locus result in both polledness and intersex conditions (Pailhoux et al. 2001). Additionally, fat-tail deposition, a key adaptive trait in sheep, has been associated with the genes *NPR2*,* HINT2*,* SPAG8*,* INSR*, both of which influence fat storage mechanisms (Ahbara et al. 2018). Chickens, which are closely related to guinea fowls, have also received considerable attention in studies aiming to understand the genetics underlying their phenotypic variations. For example, the gene *TBC1D1* (*TBC1* (tre-2/USP6, BUB2, cdc16) domain family, member 1) has been found to explain growth differences between broilers and layers (Rubin et al. 2010).

Despite the notable phenotypic variability observed in guinea fowls, specific genetic variants associated with their morphological diversity particularly variations in plumage color patterns and helmet morphology have not yet been conclusively identified. To date, research efforts have predominantly focused on descriptive phenotypic studies, as summarized in Table 3. These studies reveal substantial variation among guinea fowl populations in traits such as shank length, body length, wing length, helmet height, and live body weight, as well as in qualitative characteristics including wattle color, skin color, shank color, and overall body color. This extensive phenotypic diversity underscores the need for comprehensive genetic studies to elucidate the molecular mechanisms underpinning these traits, which could ultimately inform targeted breeding and conservation strategies.

**Table 3:** Phenotypic description of some Africa’s guinea fowl population

Country	Breed	No.	SL (cm)	BL (cm)	WL (cm)	HH (cm)	LBW (kg)	WC	SkC	SC	BC	References
Kenya	Domestic Helmeted Guinea fowls	90	7.48–8.41	45.26–48.12	18.78–20.54	2.09–2.33	1.08–1.33	Red, Blue	-	Black, Brown, White	Pearl, Lavender, White, Black	(Panyako et al. 2016)
Ghana	Guinea fowls	394	-	-	-	-	-	-	Grey, White, Dark, Yellow, Red	Black, Pink, Brown, Orange	-	(Brown et al. 2017)
Cameroon	Guinea fowls	1021	6.9–8.1	42.6–47.2	18.9–20.1	-	1.07–1.31					(Dongmo et al. 2023)
Northern Ghana	Indigenous guinea fowls	300						Red, Blue			Pearl, White, Lavender, Black	(Agbolosu et al. 2014)
Nigeria	Helmeted Guinea fowls	569	7.0-8.2	43.5–47.8	19.2–20.0	2.1–3.7	1.12–1.34	-	-	Black, Brown, White	Pearl, Lavender, White, Black	(Yakubu et al. 2022)
Nigeria	Adult Guinea fowls	-	7.3-8.0	44.0–47.0	19.0-20.3	-	1.10–1.28	-	-	-	-	(Dudusola et al. 2021)
Nigeria	indigenous guinea fowl	82	8.9	41.2	19.4	-	1.2	-	-	-	-	(Ogah 2013)

SL: shark length, BL: Body length, WL: Wing length, HH: Helmet height, LBW: Live body weight, WC: Wattle color, SkC: Skin color, SC: Shank color, BC: Body color

### Significance of guinea fowl diversity to Africa’s socio-cultural, economic and ecological landscape

Guinea fowls’ diversity exemplifies the complex interplay of cultural, economic, and ecological roles within African societies. Their importance extends beyond serving as a nutritional resource to encompass contributions to cultural practices, social cohesion, and economic resilience in rural communities across the continent. The relationships surrounding guinea fowls ranging from their use in traditional rituals and ceremonies to their role in gift exchanges and local market economies highlight their integral position within African social structures, cultural traditions, and livelihoods.

#### Economic importance

The production of guinea fowl significantly contributes to improving household livelihoods, food security, and rural economies throughout Africa. They provide essential income for rural households through the sale of live birds, meat, and eggs, with hens producing on average 80–120 eggs annually (Houndonougbo et al. 2017; Sanfo et al. 2012) which is considerably higher than the 50–60 eggs typically produced by local chickens (Amanuel et al. 2025). In many contexts, guinea fowl generate more revenue for smallholder farmers than other poultry species, particularly during the dry season when they supplement crop-based livelihoods and help alleviate poverty (Avornyo et al. 2016). Numerous smallholder farmers produce guinea fowl under semi-intensive or free-range methods, which are particularly appropriate for resource-constrained environments, as these birds require minimum feed, shelter, and veterinary care relative to other livestock species (Okyere et al. 2021; Rayan et al. 2022). Their minimal production expenses allow farmers to diversify revenue streams and engage in poultry farming with comparatively modest capital expenditure. The robust local demand for guinea fowl meat and eggs, used for domestic consumption and exchanged in local markets, enhances their economic viability and bolsters household food security (Salgado et al. 2022). In areas including northern Ghana, northern Nigeria, Sudan, and Togo, guinea fowl farming serves as a vital source of animal protein and a significant revenue stream through the sale of meat, eggs, and chicks (Madzimure et al. 2011; Mbah 2018; Abdul-Rahman et al. 2019; Suleiman and Sani 2023). Their endurance to adverse climates and capacity to flourish with few resources render them especially appropriate for small-scale and subsistence farmers, thereby enhancing rural food security, alleviating poverty, and fostering economic progress.

#### Socio-cultural importance

In addition to their economic importance, guinea fowls have a significant role in the socio-cultural frameworks of numerous African communities. In Burkina Faso, guinea fowls are significant in cultural narratives and ceremonial activities, representing protection, vigilance, and communal solidarity (Ouattara et al. 2015; Abdallah and Oluwaseun 2025). In Ghana, guinea fowls are integral to traditional folklore and ceremonies, particularly in northern communities, where they symbolize awareness, protection, and communal responsibility (Ali et al. 2019). Their social behavior, marked by robust group cohesion, is Figuratively associated with principles of communal solidarity and vigilance. Guinea fowls are integrated into numerous rites of passage, matrimonial ceremonies, festivals, and religious rituals, frequently functioning as sacrificial offerings to deities or as esteemed gifts exchanged among loved ones, thereby emphasizing their spiritual and communal significance (Abdallah and Oyebamiji 2024). This cultural integration underscores the bird’s dual function as a source of livelihood and a representation of social identity and heritage, similar to observations in certain regions of Cameroon (Massawa et al. 2020). In Zambia, guinea fowls retain significant cultural and economic importance, especially in rural communities. They are conventionally linked to characteristics such as vigilance and safeguarding and are often mentioned in regional folklore and traditional customs (Abdallah and Oyebamiji 2024). Their involvement in ceremonial exchanges and their symbolic function in strengthening social relationships highlight their significance beyond basic food security and economic generation, also acting as mediums of cultural expression and community solidarity.

#### Ecological importance

Guinea fowl *(Numida meleagris)* are increasingly recognized for their critical role in sustainable agriculture across Africa, offering both ecological and economic benefits. Under free-range systems, guinea fowls naturally forage on insects, ticks, larvae, and weeds, providing a form of biological pest control that reduces the need for chemical pesticides and herbicides (Ellis-Jones et al. 2012; Yekinni et al. 2024). Their pest-control services contribute to healthier crop production, lower input costs, and environmentally sustainable farming practices, particularly among smallholder farmers in countries such as Ghana, Nigeria, Mali, Egypt, Botswana, Zimbabwe, and Togo (Saina 2005; Negm et al. 2019; Tela et al. 2021). In addition, guinea fowl manure is valued for enhancing soil fertility through the deposition of nutrient-rich droppings (Juba 2011; Massawa et al. 2023; Douan et al. 2024; Oguche et al. 2025). Their manure acts as an organic fertilizer, enriching soil quality, boosting microbial activity, and improving soil structure, thereby reducing reliance on synthetic fertilizers. In areas with harsh climates and resource-constrained farming systems such as Sudan, the Sahel region, and parts of Egypt and Botswana the resilience and low-input requirements of guinea fowls make them particularly suitable for sustainable agriculture (Moreki and Seabo 2012; Negm et al. 2019; Molina-Flores et al. 2020). Their ecological functions align with broader agro-ecological and climate-resilient farming practices needed to enhance food security and biodiversity conservation. Beyond pest control and soil improvement, guinea fowls contribute to ecological balance by promoting biodiversity in mixed farming systems, enhancing organic farming efforts, and reducing environmental degradation (Darkoh 2003; Tela et al. 2021; Niba 2025). Their incorporation into traditional and smallholder agricultural systems across Africa particularly in Ghana, Cameroon, Togo, and Zimbabwe supports long-term food security, rural economic development, and environmental protection (Saina 2005; Sanginga and Woomer 2009; Pervarah et al. 2023).

### The proposed future direction for sustainable breeding and utilization of Africa’s guinea fowls

Despite their adaptability to harsh climatic conditions and resistance to major poultry diseases, guinea fowl productivity remains low due to limited investment in management practices, selective breeding, and market development (Yaro 2016). Given the growing need for diverse sources of animal-based protein, guinea fowl production presents a significant opportunity to enhance food security and rural livelihoods (Abdallah and Oluwaseun 2025). However, before developing strategic interventions to promote sustainable production and conservation particularly in the context of climate change, it is essential to first explore and understand the challenges that impede their effective production.

#### Challenges impending guinea fowl breeding in Africa

**Fig. 2:**
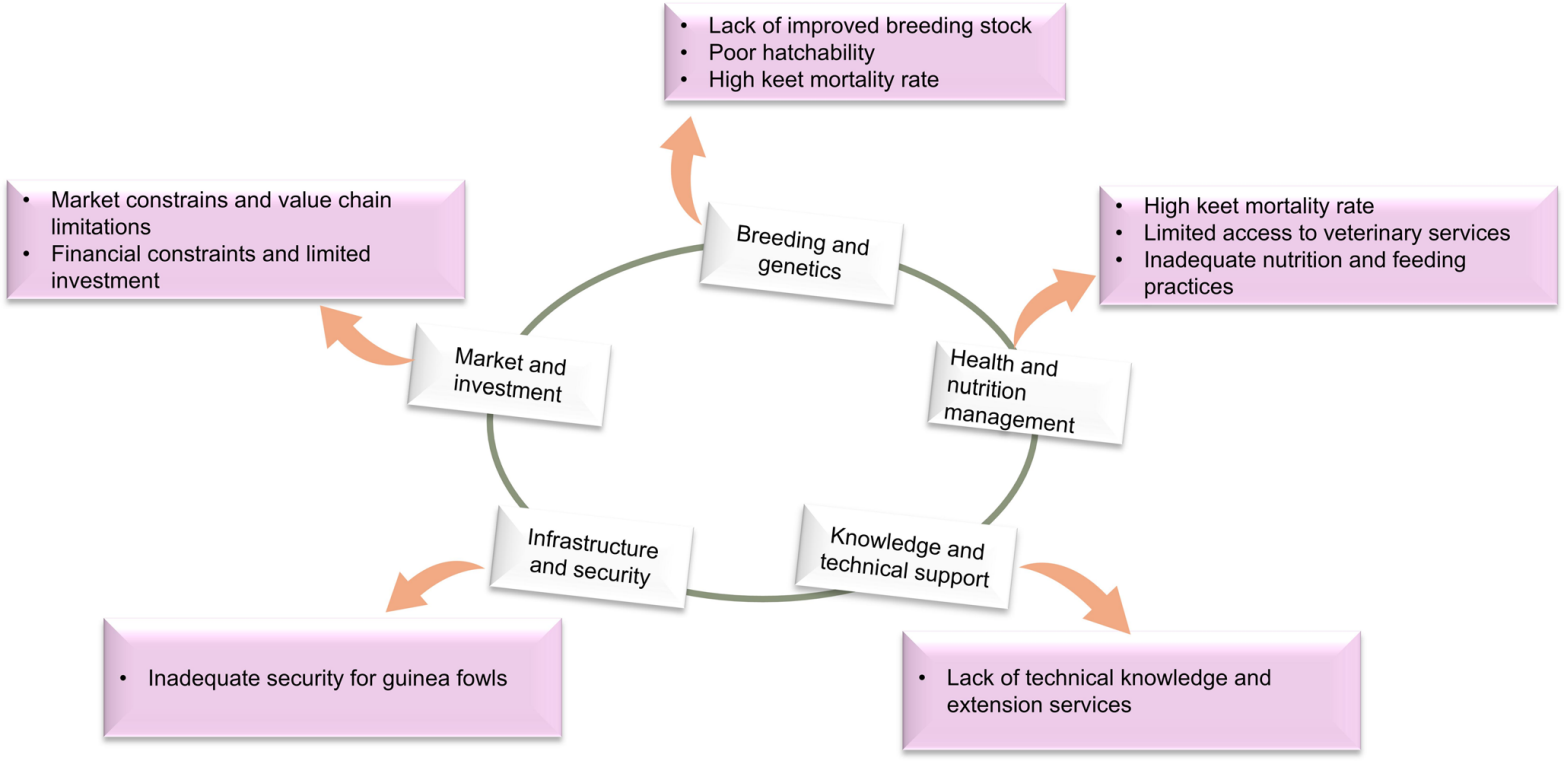
Challenges affecting sustainable breeding and utilization of guinea fowls in Africa

The challenges hindering the sustainable production and utilization of guinea fowls can be categorized into five major groups, as illustrated in Fig. 2. These challenges have been documented across various studies (e.g., Abdallah and Oluwaseun 2025; Abdallah and Oyebamiji 2024; Gono et al. 2013; Moreki and Radikara 2013), spanning different countries and highlighting(Gono et al. 2013; Moreki and Radikara 2013; Abdallah and Oyebamiji 2024; Abdallah and Oluwaseun 2025) persistent trend affecting this important poultry sub-sector.

- Lack of genetically improved breeding stock

One of the major challenges in guinea fowl production is the lack of improved genetic stock. Most populations remain unimproved, resulting in slow growth rates, low egg production, and wide variability in performance. Genetic improvement can be achieved by selecting superior breeds and developing controlled hybrids. Selective breeding programs that focus on traits such as early maturity, rapid growth, high egg production, and feed efficiency could significantly boost productivity. Controlled crossbreeding can produce birds with enhanced performance while maintaining adaptability to local conditions. At the same time, conserving indigenous genetic resources is vital for maintaining biodiversity and securing material for future breeding efforts (Moreki and Radikara 2013; Houndonougbo et al. 2017).

b. Inadequate security for guinea fowls

In many African countries, guinea fowl production is predominantly practiced under free-range systems (Melesse 2014). While this method requires minimal input, it exposes birds to several risks, including predation by stray dogs, theft, and harsh environmental conditions (Avornyo et al. 2016). Inadequate housing structures further exacerbate these vulnerabilities, leading to significant losses in production (Avornyo et al. 2016). Housing is one aspect of guinea fowl production that may require relatively high capital investment, which can be challenging for rural families. As a result, many farmers continue to rely on traditional houseless management systems. However, transitioning from purely extensive systems to semi-intensive management could lead to significant improvements in productivity by reducing losses and enhancing growth performance. For instance, studies have shown that insufficient shelter contributes to high rates of predation and mortality among guinea fowl populations (Saina 2005). To address these challenges, the introduction of modern, affordable, and climate-adaptable housing systems using locally available materials is recommended. Such innovations would enhance the survival and productivity of guinea fowls while remaining accessible to smallholder farmers, thereby strengthening rural livelihoods and food security.

c. High keet mortality rate

Another most critical challenge in guinea fowl production is the high mortality rate of keets. A survey conducted byKouassi et al. (2019) in Ivory coast showed that, the major concern by guinea fowl farmers was mortality rate of young guinea fowl keets. Factors such as exposure to harsh weather conditions, inadequate feeding, poor nutrition, and the lack of proper brooding facilities contribute significantly to these high mortality rates. Additionally, the absence of reliable sources for quality day-old keets further hampers efforts to improve flock sizes and overall productivity. Inadequate availability of high-quality day old keets for development of breeding stock is highly associated with poor hatchability of guinea fowls’ eggs possibly due to the characteristics of its eggshell (Dahouda et al. 2007; Kouassi et al. 2019; Adu-Aboagye et al. 2020). Further, since guinea fowl cocks practice monogamy mating behavior and hence, some guinea fowl hens might lay unfertilized eggs. To address these challenges, it is essential to improve access to veterinary services, implement effective vaccination programs, and promote the construction of predator-proof housing. Additionally, farmer training on guinea fowl breeding management particularly during the breeding season can enhance their capacity to handle hatching and mating behaviors effectively, thereby improving productivity and flock survival.

d. Inadequate nutrition and feeding practices

According to Ebegbulem (2018), guinea fowls have specific nutritional requirements that differ significantly from the conventional requirements of chickens. However, many farmers rely primarily on scavenging systems and provide little to no supplementary feeding. In some cases, farmers use feed formulations designed for chickens, which often fail to meet the higher protein requirements of guinea fowls (Moreki and Radikara 2013). Such feeding practices result in poor or slow growth rates, low egg production, and increased susceptibility to diseases. Furthermore, the unavailability of commercially formulated feeds specifically for guinea fowls exacerbates nutritional challenges (Abdallah and Oyebamiji 2024). To overcome these issues, farmers should be trained to formulate and balance rations using locally available feed resources tailored to the nutritional needs of guinea fowls.

e. Lack of technical knowledge and extension services

There is limited information available on best practices for guinea fowl rearing (Abdallah and Oyebamiji 2024). Many farmers lack access to extension services that could provide essential guidance on housing, feeding, breeding, disease prevention, and overall flock management (Swanson 2008). Although many African countries have made efforts to strengthen agricultural extension services, the delivery methods often remain outdated and analog, resulting in low coverage and adoption rates among farmers. Incorporating modern technology (Khan et al. 2025) into extension systems is essential to broaden outreach and engage innovative youth in agriculture. The knowledge deficit leads to substandard management practices, ultimately reducing profitability and productivity. To address this, training programs should be implemented to educate farmers on the importance of biosecurity measures, improving overall flock health and production efficiency.

f. Limited Access to veterinary services

Although adult guinea fowls are relatively resistant to common poultry diseases such as coccidiosis and Newcastle disease, their keets are highly vulnerable (Moreki and Radikara 2013). The unavailability of veterinary services, particularly in rural farming communities, increases the risk of disease outbreaks, which can decimate entire flocks (Ebegbulem 2018). Moreover, the widespread local belief that guinea fowls are inherently disease-resistant often leads farmers to neglect essential preventive measures such as vaccination and deworming. This lack of adherence to preventive health practices significantly contributes to high mortality rates among guinea fowls. To some extent, farmers self-administer antibiotics to sick birds rather than seeking professional veterinary services. For instance, a study in Togo by Tcheou et al. (2025) reported a 49.43% incidence of antibiotic residues in guinea fowl eggs, highlighting the risks associated with unregulated antibiotic use. To address these challenges, it is recommended to establish mobile veterinary clinics and rural distribution points to improve service accessibility. Additionally, farmers should be trained in the importance of practicing strict biosecurity measures to enhance flock health and survival rates.

g. Poor hatchability and breeding challenges

Guinea fowl exhibit low hatchability rates, which are frequently attributed to inadequate incubation practices and a lack of or limited knowledge regarding effective breeding techniques (Araújo et al. 2023). The reliance on natural incubation methods, combined with environmental factors, contributes to poor and inconsistent hatching success (Abdallah and Oyebamiji 2024). Furthermore, the absence of well-structured breeding programs designed to enhance genetic stock limits the potential for improving productivity (Houndonougbo et al. 2017). Another key challenge affecting hatchability is the nature of the eggshell, as previously discussed. Additionally, many guinea fowl hens may lay unfertilized eggs due to the species’ natural monogamous mating behavior, where a male often mates exclusively with a single female, limiting fertilization rates in larger flocks. Consequently, the promotion of improved breeding techniques and selective breeding practices is essential for addressing these challenges.

h. Market constraints and value chain limitations

Guinea fowl product commercialization is constrained by poorly developed market structures and value chains (Abdul-Rahman et al. 2019). Typically, guinea fowl products are sold through informal channels, which limits market access and price stability for producers, coupled with limited awareness of their nutritional value (Kwesisi et al. 2022). Furthermore, the lack of modern processing facilities and marketing strategies by farmers reduces the competitiveness of guinea fowl products in both local and international markets (Moreki and Radikara 2013). Developing local and regional markets, along with a cooperative marketing system, is essential to overcoming market access and value addition challenges in guinea fowl production. For example, adopting a “from farm to fork” strategy similar to that implemented in the European Union (Abbasi et al. 2023) or designing a specialized product value chain like the one established for the Mangalica pig in Hungary (Pocsai 2012), can enhance marketability, traceability, quality assurance, and consumer confidence, while boosting income for producers.

i. Financial constraints and limited investment

Generally, farmers in many African countries face significant challenges in accessing credit facilities to support their agricultural activities. In the case of guinea fowl production, these difficulties are further compounded by the fragmented and informal nature of the market infrastructure, making it even harder for producers to secure financial services tailored to their needs. In the absence of sufficient funding, farmers face difficulties in financing improved housing, nutritious feed, and proper healthcare for their flocks. This financial limitation perpetuates a cycle of low productivity and restricts the ability to scale up guinea fowl production (Shoyombo et al. 2021). This challenge is exacerbated by the lack of a well-structured guinea fowl value chain, which subjects the industry to market uncertainty. Although there has been a surge in digital lending platforms (Choruma et al. 2024), these services are often expensive and offer limited repayment periods (Sommer 2021), which many guinea fowl farmers may find difficult to manage. Therefore, intentional provision of financial support to guinea fowl farmers, along with the establishment and strengthening of a guinea fowl product-specific value chain, would enhance production and contribute to the conservation of guinea fowls.

#### Proposed conceptual framework for sustainable production and conservation of Africa’s guinea fowls

**Fig. 3:**
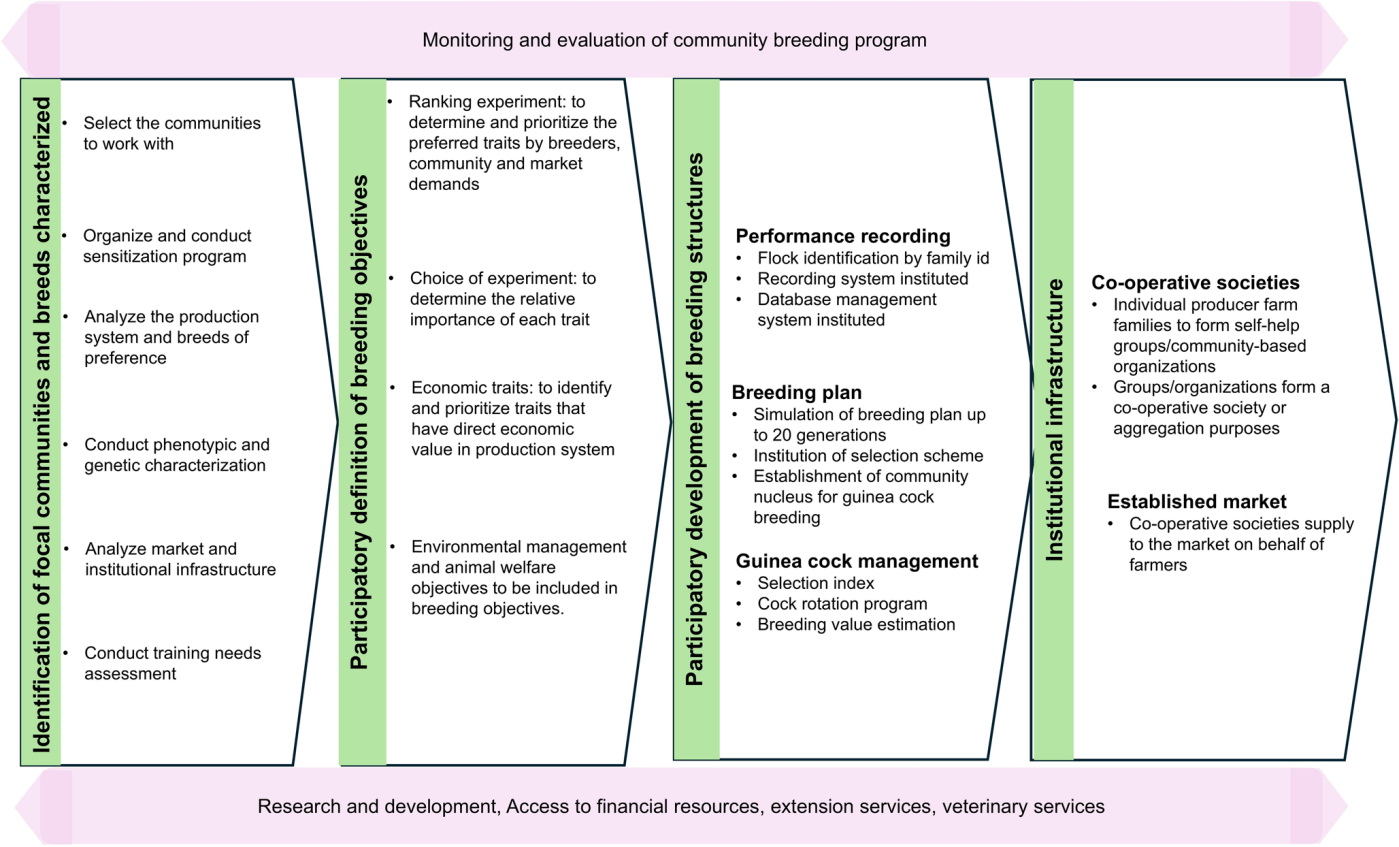
Proposed guinea fowl community breeding program in Africa

Guinea fowl production in Africa faces multifaceted challenges that span genetics, health, management, market access, and finance. These issues are deeply interconnected and cannot be effectively addressed in isolation. Therefore, a multi-sectoral approach is essential. However, even with such collaboration, productivity will remain low without addressing the foundational issue of genetics. To this end, we propose the establishment of a community-based breeding program that focuses on the development and dissemination of improved genetic stock (Fig. 3). This program should be coupled with intensive capacity building for local breeding communities to ensure sustainability and proper management. Additionally, the creation of a specialized guinea fowl product value chain will be critical. By targeting premium markets, this value chain can significantly boost farmers’ incomes and make guinea fowl farming a more viable and attractive livelihood option. Effective agricultural marketing is frequently facilitated through the aggregation of farm outputs. In the context of African agricultural systems, cooperative societies have demonstrated considerable efficacy in consolidating produce from smallholder farmers and managing collective sales (Cheyo et al. 2024). This aggregation strategy enables farmers to leverage economies of scale, thereby enhancing their bargaining power, reducing transaction costs, and improving market access. Consequently, farmers engaged in cooperative marketing structures often realize higher returns compared to those who operate individually.

## Conclusion

The present study highlights the global neglect of Africa’s guinea fowl sub-sector, despite its considerable potential to alleviate poverty and enhance food security in rural communities—particularly in the face of increasing climate variability. Through a comprehensive literature review, we demonstrate that guinea fowls not only offer economic benefits but also possess substantial socio-cultural and ecological significance. These multifaceted roles are closely linked to the species’ phenotypic variation, which is ultimately underpinned by genetic diversity. Accordingly, there is a critical need to develop strategies that promote the sustainable breeding, utilization, and conservation of guinea fowls. To address this, we have proposed a community-based breeding program that could serve as a strategic framework for policymakers and breeding organizations aiming to establish sustainable breeding initiatives for this important avian species. To enhance guinea fowl breeding in Africa, it is necessary to invest in research on economically important traits and the best strategies to improve them for the benefit of the community.

## Data Availability

The data that support the findings of this study are available from the corresponding author upon request.
